# A Question of Damages

**Published:** 1902-05

**Authors:** 


### A Question of Damages.

THERE has been decided in New York City a case which has a peculiar interest in Buffalo inasmuch as it will affect the question of the payment of damages by two Buffalo hospitals.

Something over two years ago Helen G. Ward was taken to St. Vincents' Hospital for operation. At the conclusion of surgical procedures she was placed in bed and a nurse in charge of the case put hot water bags about her. She suffered burns more or less severe from these hot water bags. When she left the hospital she brought suit for damages against the hospital and was by direction of the court non-suited. An appeal resulted in a new trial being ordered in which case the jury disagreed. A third trial was secured and the jury gave Miss Ward a verdict of $10,000. The hospital's attorneys prepared papers in appeal and carried up the case. The Appellate Division set aside the $10,000 verdict and ordered a new trial on errors. The case was tried before Justice Beach, in New York City, and resulted in an increased verdict of $18,000 for Miss Ward.

The hospital authorities will now probably pay the verdict for, according to the opinions of Buffalo lawyers who have been watching the progress of this remarkably well-fought case, there is nothing on which to base another appeal, the last case having been tried with a rigid observance of the rules of evidence, and great care having been exercised by the trial judge with a view to preventing either side from taking advantage of any errors of judgment. The main contention of the hospital was to the effect that all necessary care was exercised in the treatment of the patient; that the burns were unavoidable. The plaintiff set up that the nurse in charge of her case after the operation was an undergraduate and therefore not wholly qualified to act. A novel proposition raised by the hospital's attorneys was that the placing of a hot water bag did not require any special degree of qualification, hence the question of inexperience was not to be raised. The jury appears to have held. however, that the nurse was an agent of the hospital and that the latter was therefore liable for her acts.

There are two similar cases in Buffalo which will now either be brought to trial or settled on the strength of this verdict. In each case patients were burned more or less severely by the application of hot water bottles placed by undergraduate nurses. In one case it is stated that considerable deformity has resulted.

---

CHRISTIAN science has had another seance with the coroner in Buffalo. Fortunately for the highly respected society leader who in this case was the healer, the patient's death was due to carcinoma of the stomach for which the healer gave treatments, absent and contact, without, however, having an effect on the progressive cancer, and the patient died in great agony. The family reported the case to the coroner's office and a postmortem revealed the cause of death. It also showed that the patient's life might have been prolonged for a considerable period had she not been blinded by the glitter of a false faith and the unctious and christian-like promises of a society woman, who has forsaken the delights of the pink tea for the more lucrative life of bewildering the really sick with the false doctrines of mind and matter as applied to the treatment of human ills.

